# AI-powered remote monitoring of brain responses to clear and incomprehensible speech via speckle pattern analysis

**DOI:** 10.1117/1.JBO.30.6.067001

**Published:** 2025-06-09

**Authors:** Natalya Segal, Zeev Kalyuzhner, Sergey Agdarov, Yafim Beiderman, Yevgeny Beiderman, Zeev Zalevsky

**Affiliations:** a Bar-Ilan University, Faculty of Engineering and the Nanotechnology Center, Ramat-Gan, Israel; b Holon Institute of Technology, Faculty of Electrical and Electronics Engineering, Holon, Israel

**Keywords:** remote brain monitoring, laser speckle patterns, AI-driven neuroimaging, photonic brain sensing, speech response detection, noninvasive brain analysis, Wernicke’s area

## Abstract

**Significance:**

Functional magnetic resonance imaging provides high spatial resolution but is limited by cost, infrastructure, and the constraints of an enclosed scanner. Portable methods such as functional near-infrared spectroscopy and electroencephalography improve accessibility but require physical contact with the scalp. Our speckle pattern imaging technique offers a remote, contactless, and low-cost alternative for monitoring cortical activity, enabling neuroimaging in environments where contact-based methods are impractical or MRI access is unfeasible.

**Aim:**

We aim to develop a remote photonic technique for detecting human brain cortex activity by applying deep learning to the speckle pattern videos captured from specific brain cortex areas illuminated by a laser beam.

**Approach:**

We enhance laser speckle pattern tracking with artificial intelligence (AI) to enable remote brain monitoring. In this study, a laser beam was projected onto Wernicke’s area to detect brain responses to a clear and incomprehensible speech. The speckle pattern videos were analyzed using a convolutional long short-term memory–based deep neural network classifier.

**Results:**

The classifier distinguished brain responses to a clear and incomprehensible speech in unseen subjects, achieving a mean area under the receiver operating characteristic curve (area under the curve) of 0.94 for classifications based on at least 1 s of input.

**Conclusions:**

This remote method for distinguishing brain responses has practical applications in brain function research, medical monitoring, sports, and real-life scenarios, particularly for individuals sensitive to scalp contact or headgear.

## 1 Introduction

Human brain research seeks to enhance understanding of brain function, improve diagnostics for conditions such as stroke and psychiatric disorders,^1^ and drive innovations such as brain–machine interfaces.^2^^–^^4^ Early observations of brain injuries, documented in the Edwin Smith Surgical Papyrus (16th century BC) and by Avicenna (980 to 1037 CE), laid the groundwork for modern advancements. Today, technological progress has prioritized accessibility, precision, and noninvasiveness in brain research. Optical techniques, in particular, have enabled portable and contactless methods for studying brain activity, paving the way for innovative imaging technologies, such as the one presented in this study.

### 1.1 Noninvasive Methods for Functional Brain Research

Noninvasive methods for functional brain research include electroencephalography (EEG), functional magnetic resonance imaging (fMRI), magnetoencephalography (MEG), and functional near-infrared spectroscopy (fNIRS).^5^^–^^8^ fMRI provides whole-brain imaging with high spatial ($∼1\;\;mm^{3}$) but lower temporal (∼1  s) resolution by detecting blood flow variations using the magnetic properties of hemoglobin and blood oxygen level–dependent contrast to map neural activity.^9^ MEG measures magnetic fields generated by neuron groups (∼50,000 to 100,000), offering ∼2 to 3 mm spatial resolution and detecting activity beyond the cortex in both active and sleeping states.^1^^,^^7^ EEG records electrical activity with high temporal (∼1  ms) but low spatial resolution, requiring electrodes in contact with the scalp.^5^^,^^10^ fNIRS, an optical method, uses near-infrared light (700 to 1000 nm) to measure hemodynamic changes at the cortical surface with ∼2 to 3 cm spatial resolution, offering portability and motion tolerance, though it also requires physical contact and baseline measurements of oxyhemoglobin (oxy-Hb) and deoxyhemoglobin (deoxy-Hb) concentrations from a resting state.^8^^,^^11^^–^^13^ Each method has limitations: fMRI and MEG are costly, stationary, and sensitive to motion, whereas EEG and fNIRS, though portable, are restricted to cortical activity and require physical contact. In addition, fNIRS signals are influenced by skin and hair properties.^1^^,^^8^^,^^10^ These limitations underscore the need for a portable, noncontact method that enables reliable brain monitoring in dynamic environments. Speckle imaging approaches offer a promising path toward addressing these challenges and advancing brain imaging technology.^14^^–^^17^

Speckle-based optical methods such as speckle contrast optical spectroscopy, speckle visibility spectroscopy, and diffuse speckle contrast analysis have emerged as promising tools for brain monitoring, enabling noncontact measurement of cerebral hemodynamics and stroke risk via portable systems.^18^^–^^20^ Unlike contrast imaging, which captures speckle patterns in the focused plane and detects flow via contrast variation, our method captures secondary speckle patterns in the defocused plane, enabling the detection of surface microvibrations linked to cortical activity. This approach requires less tissue penetration and provides rich 2D spatial information per frame, enhancing the performance of deep learning analysis.

### 1.2 Human Brain Response to Sound and Speech

Noninvasive methods have been widely used to investigate brain responses to sound and speech stimuli. Key areas involved in language processing include the primary auditory cortex, Wernicke’s area, and Bruce’s area.^21^^–^^35^ Wernicke’s area [Brodmann area (BA) 22], essential for speech comprehension, is located in the left hemisphere in 95% of right-handed and 70% of left-handed individuals.^32^ Studies using fMRI highlight the temporal dynamics of language processing. Semantic and syntactic relations are processed between 300 and 500 ms after stimulus onset, involving Wernicke’s area and frontal regions such as BA 45 (semantic processing) and BA 44 (syntactic processing).^35^ Integration of these processes occurs ∼600  ms after stimulus input, reflecting the brain’s ability to handle complex language structures.

Despite the utility of traditional neuroimaging methods such as fMRI and EEG, they present challenges, including high costs and the need for physical contact. To address these limitations, this study employs an innovative optical approach leveraging secondary speckle pattern analysis to remotely monitor brain cortex activity.

### 1.3 Human Brain Activity Secondary Speckle Patterns Analysis

Methods based on recording and processing speckle patterns reflected from a rough surface illuminated by a laser beam^17^^,^^36^ have been successfully used in engineering^37^^,^^38^ for several decades and also impacted medical diagnostics.^39^^–^^42^ Recent research^43^ represents the first step toward adopting this technique for recording human brain cortex activity, particularly in response to auditory stimuli. It was found that physiological processes associated with hemodynamic brain activity due to auditory stimulation of the human cerebral cortex could be identified by remotely monitoring nanovibrations in response to transient blood flow to specific brain regions. One study^43^ accurately classified present and absent auditory stimulation by recording and processing videos of the speckle patterns reflected from specific brain cortex areas illuminated by a laser beam. These videos were then analyzed using deep learning algorithms.

### 1.4 Purpose of this Study

This study aimed to distinguish brain responses to clear and incomprehensible speech by analyzing secondary speckle patterns reflected from targeted scalp areas. By integrating this optical technique with artificial intelligence (AI)-based data analysis [in particular deep neural network (DNN) based on convolutional long short-term memory (ConvLSTM)], we seek to establish a novel, affordable, and portable method for remote, noninvasive brain cortex activity monitoring. This approach has the potential to advance neuroscience research, aid in diagnosing cortical damage, and support applications such as brain–computer interface technology.

## 2 Materials and Methods

### 2.1 Theoretical Background—Secondary Speckle Patterns

When a laser beam interacts with a rough surface and undergoes back-scattering, it induces a random phase distribution in the far field, forming self-interfering secondary speckle patterns.^36^

**Fig. 1:**
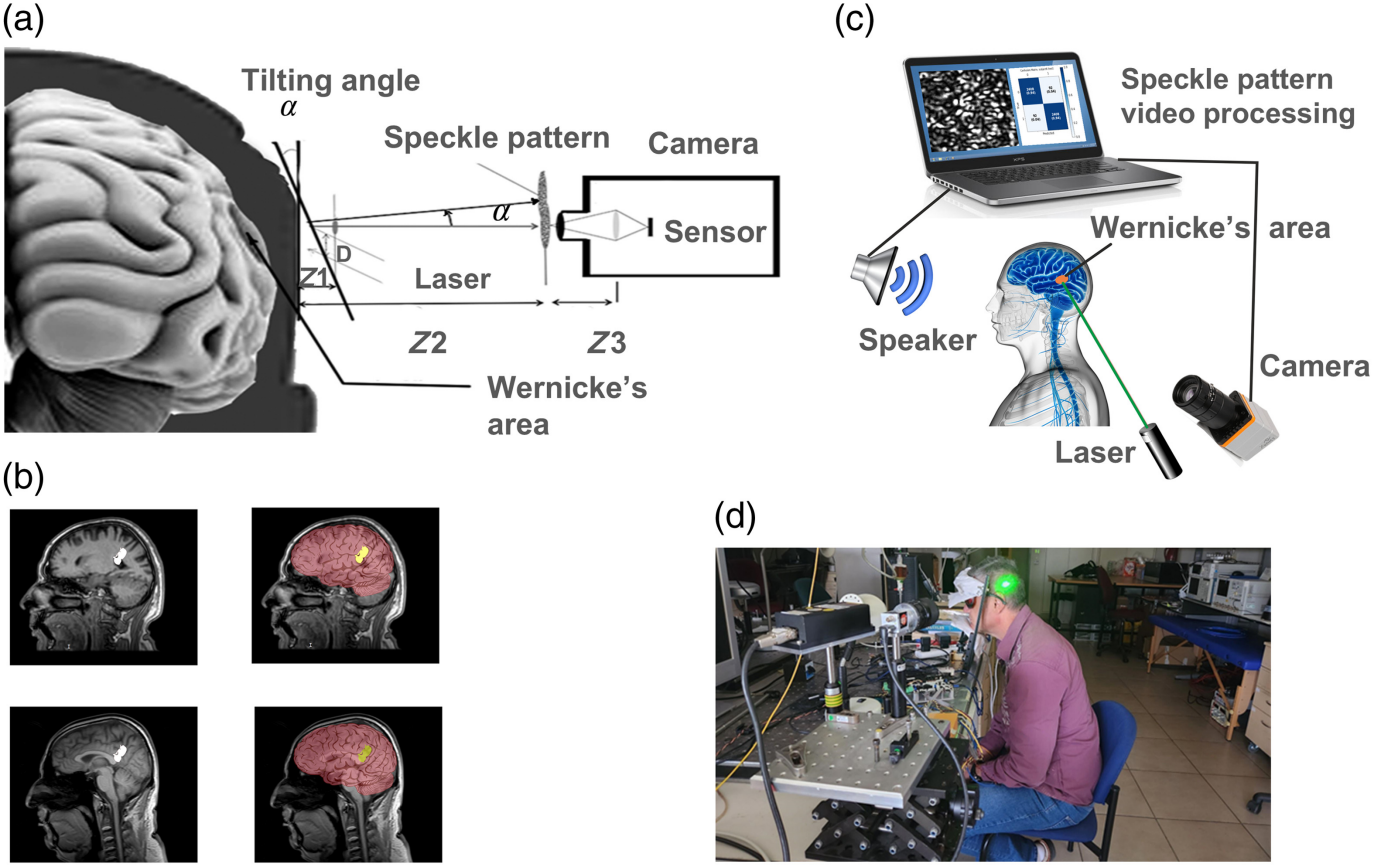
(a) Schematic illustration of the system used to capture secondary speckle patterns reflected from Wernicke’s area.^17^ (b) MRI scans of the left cortical hemisphere in two subjects (subjects 5 and 6) were used to localize Wernicke’s area. The left panels highlight Wernicke’s area in white, whereas the right panels overlay additional schematic outlines of relevant cortical regions. The top row corresponds to subject 5, and the bottom to subject 6. (c) A schematic view of the experimental setup. (d) A photograph provides a general view of the setup.

The vibration of the illuminated surface causes lateral shifts in the secondary speckle patterns recorded by a defocused camera (defocus creates the far-field effect). Depending on the movement of the illuminated surface, the speckle pattern experiences temporal displacements (shifts) proportional to the tilt of the surface.^19^ The correlation among the tilt angle α of the illuminated surface, the laser wavelength λ [Fig. 1(a)], and the linear phase factor β can be expressed as follows:^17^

$$\beta =\frac{4\pi \;\tan\;\alpha }{\lambda }≃\frac{4\pi \alpha }{\lambda }.$$ (1)

The requirement for the focal length (F) of the camera used to generate the speckle pattern is expressed in Eq. (2) and Ref. 37

$$F=\frac{K\Delta xZ_{3}D}{Z_{2}\lambda },$$ (2)

where K is the minimum number of pixels in which every speckle in the detector plane is observed, Δx is the size of the pixel in the detector, $Z_{2}$ is the distance between the illuminated surface and the secondary speckle plane formation (due to defocusing), and $Z_{3}$ is the distance between the secondary speckle pattern plane formation and the camera lens. D is the diameter of the laser spot. The distance $Z_{2}$ is required to fulfill the far-field approximation as follows:

$$Z_{2}>\frac{D^{2}}{4\lambda }.$$ (3)

The phase factor $\beta =[\beta_{x},\beta_{y}]$, introduced in Eq. (1), is a linear function of the surface tilt angles $\alpha =[\alpha_{x},\alpha_{y}]$ for a given wavelength. It defines the phase gradient in the transverse (x,y) plane and is proportional to the local angular tilt of the illuminated scalp surface. This parameter characterizes the relationship between the original surface roughness and the speckle pattern captured by the camera. The recorded speckle image arises from a combination of static surface roughness, tilt-induced phase shifts (parameterized by β), and far-field diffraction, which converts these phase variations into measurable displacements in the transverse plane,^37^ enabling the application of machine learning algorithms on the recorded speckle pattern videos to extract brain activity-related features from speckle pattern dynamics.

### 2.2 Theoretical Background—Methods of the Speckle Patterns Analysis

We assume that speckle pattern videos, captured from a specific scalp area illuminated by a laser beam, contain information about the human brain’s cortex response to stimuli. A laser could be directed at the scalp area above Wernicke’s zone of the brain cortex, which is related to speech comprehension.

Speckle patterns recorded by a defocused camera consist of a sequence of frames differing by horizontal and vertical shifts. Cross-correlation of consecutive frames^17^ allows the transfer of information to a displacement graph, which can be further analyzed by traditional methods or by AI. Cross-correlation is a conventional method for analyzing speckle pattern videos and has proven effective for remote optical heartbeat measurements^41^ and many other applications.

The development of modern DNN architectures, coupled with the sharp increase in hardware capacity and speed, enables direct analysis of secondary speckle pattern videos by DNNs.^43^ A DNN comprises an input layer, hidden layers, and an output layer. During training, parameters are optimized, and hyperparameters are fine-tuned during validation. Our task was framed as binary classification, with the output layer assigning probabilities to the clear or incomprehensible language classes. In our case, the network’s input data unit is a chunk of a speckle pattern video with an arbitrarily selected number of frames per chunk. The nature of the input data is both spatial and temporal, which suggests the use of ConvLSTM in the hidden layers.^44^ ConvLSTM combines the properties of convolutional neural networks and long short-term memory (LSTM) in the same layer. An additional motivation to consider ConvLSTM is related to the results obtained by a similar study.^43^

### 2.3 Theoretical Background—ConvLSTM

The ConvLSTM layer combines the advantages of an LSTM and a convolutional layer, capturing spatial and temporal information.^44^ The key equations of the ConvLSTM cell are similar to those of a regular LSTM and differ only in terms of the convolutional operators (*) instead of regular multiplications:

$$i_{t}=\sigma (W_{xi}*X_{t}+W_{hi}*H_{t-1}+W_{ci}\cdot C_{t-1}+b_{i}),$$ (4)

$$f_{t}=\sigma (W_{xf}*X_{t}+W_{hf}*H_{t-1}+W_{cf}\cdot C_{t-1}+b_{f}),$$ (5)

$$o_{t}=\sigma (W_{xo}*X_{t}+W_{ho}*H_{t-1}+W_{co}\cdot C_{t}+b_{o}),$$ (6)

$$C_{t}=f_{t}\cdot C_{t-1}+i_{t}\cdot \tanh(W_{xc}*X_{t}+W_{hc}*H_{t-1}+b_{c}),$$ (7)

$$H_{t}=o_{t}\cdot \tanh(C_{t}).$$ (8)

**Fig. 2:**
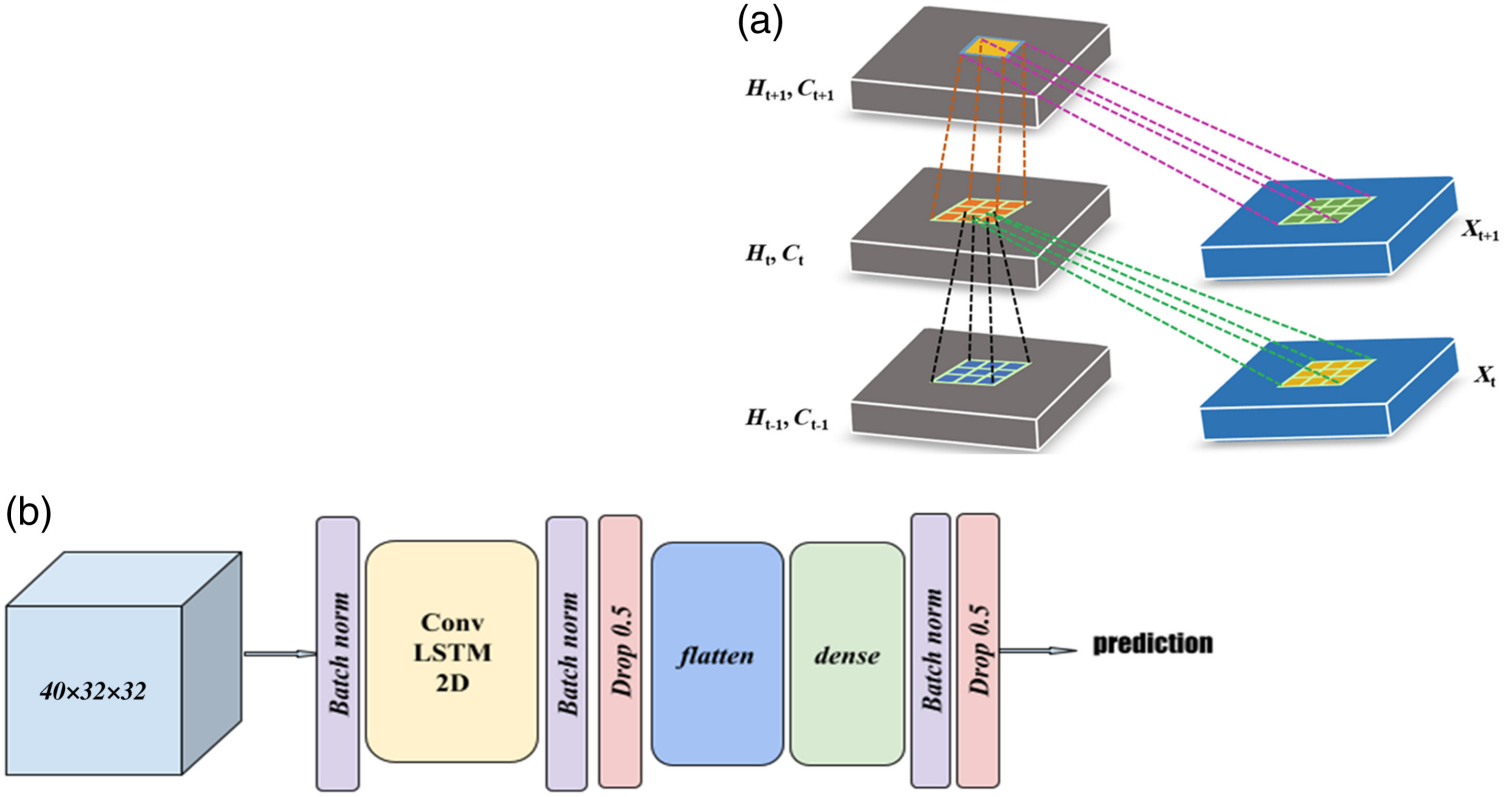
(a) The inner structure of ConvLSTM 2D layer. (b) Layers of the proposed DNN model. The input is a speckle pattern video divided into 40-frame chunks, where every frame resolution is 32×32, and the output is a probability that the input belongs to the first class (in English). Classes are defined according to the auditory stimulation that the subject receives.

In Eq. (4), $H_{t}$ is the cell output, $C_{t}$ is the cell state (a memory cell), * stands for the convolutional product, and “·” represents the elementwise product, $X_{1},\ldots ,X_{t}$ are inputs, and $i_{t}$, $f_{t}$, and $o_{t}$ are the gates of the ConvLSTM (input, forget, and output, respectively). Gates $i_{t}$, $f_{t}$, and $o_{t}$ of the ConvLSTM are 3D tensors, the last two dimensions of which are spatial (rows and columns). To better describe the inputs and states of ConvLSTM, one can imagine them as vectors standing on a spatial grid. ConvLSTM determines the future state of a specific cell in this grid based on the inputs and past states of its neighboring cells. This is accomplished using a convolution operator for state-to-state and input-to-state transitions^44^ [Fig. 2(a)].

We used the adaptive moment estimation (Adam) algorithm as the optimization technique for gradient descent.^45^ The sigmoid function Eq. (9), where x is the input tensor for the last layer, was applied to output probabilities within the range [0, 1] for binary classification

$$f(x)=\frac{1}{1+e^{-x}}.$$ (9)

### 2.4 Experimental Setup

To carry out remote sensing of human brain activity under specific sound stimulation, we assembled a setup containing the following components: a support to fix the tested human head, a laser illuminating the selected skull area, a digital camera capturing the secondary speckle patterns reflected from the head, a loudspeaker broadcasting the selected sound recordings, and a computer saving and processing the recorded video files using an AI-based algorithm [see Figs. 1(c) and 1(d)].

We used a 532-nm green LaserPPGL-210F CW 300 mW and a Basler camera (caA1440-220  μm) equipped with a Computar lens (f55 mm) set to defocus. Defocusing the camera allowed us to reach the effect of a far-field within a small distance from the illuminated surface. In the far field, vertical and horizontal tilt are captured as vertical and horizontal shifts (respectively), making it easier to process. The exposure time was 700  μs. The setup distances were $Z_{2}=200\;\;\mathrm{mm}$ (from the laser-illuminated surface to the speckle plane) and $Z_{3}=55\;\;\mathrm{mm}$ (lens focal length). The original speckle size was ∼16  pixels in the 128×64  pixel images, providing sufficient visibility for analysis after downsampling to 32×32  pixels. Downsampling was performed using bilinear interpolation, which maintains the speckle pattern’s motion dynamics despite minor contrast blending at the speckle edges.

A 532-nm green laser (LaserPPGL-210F CW, 300 mW) was selected over near-infrared alternatives to enhance surface contrast and avoid double-speckle patterns caused by overlapping reflections from different tissue layers. The visible wavelength also allowed precise visual placement of the laser spot on the scalp, simplifying alignment. The laser was positioned ∼20  cm from the subject’s head to ensure adequate illumination and convenient targeting. This distance was chosen for experimental practicality, but it is not critical; similar results can be obtained at other distances, provided the speckle quality and illuminated area are maintained. The collimated laser beam produced a ∼1  cm spot on the scalp, yielding a power density of $∼0.057\;\;\mathrm{mW}/mm^{2}$ (based on 4.5 mW over an area of $∼78.5\;\;mm^{2}$).

The experiments were conducted under stable and reproducible conditions in a controlled laboratory environment. This included a fixed optical setup, the absence of direct daylight, and the minimization of subject motion. Participants were instructed to remain still during recordings. Hair movement was restricted using hairpins to reduce interference with the laser beam.

The laser placed ∼20  cm from the tested head, illuminated Wernicke’s area, which is related to speech comprehension. Eight participants aged 26 to 77 were involved in the tests, conducted over several days. Each participant signed a written informed consent form to participate in the study, and IRB approval (the approval number is 290425572) was obtained from the Human Subjects Institutional Review Board (HSIRB) at Bar-Ilan University. All procedures were conducted in accordance with the principles of the Declaration of Helsinki. Participants also gave their written permission to use their photographs and MRI scans in the paper.

To approximate the location of Wernicke’s area, we overlapped the MRI scans of two participants with the corresponding profile images. The area on the participant’s head was marked before the test [Fig. 1(b)]. Based on the provided measurements, we also approximated the area location for the remaining participants.

The head of each tested participant was fixed on a support to minimize voluntary movements. The left side of the head was illuminated by a laser beam, touching the scalp area of interest above Wernicke’s area. The experiments were conducted in a controlled laboratory environment. Subjects were asked not to move during the recording. Hair movements were restricted, and external noise was minimized. Although the laser power was set below the eye-safety level of 1 mW, the participants wore protective glasses [see Fig. 1(d)].

The following scenarios were generated, and videos containing speckle patterns reflected from the human head were consequently recorded: English (clear for all participants) and Swedish (incomprehensible). To ensure a controlled comparison of linguistic stimuli, we employed monologue speech samples from the same speakers, thus maintaining consistent speaker characteristics across both stimulus types. Both speakers were nonprofessional video bloggers of the same gender and similar age. The two chosen languages share comparable features, including pause rates. The clear speech was in a language familiar to participants, whereas the incomprehensible speech was in a language none of the participants understood.

The Basler camera was set to a resolution of 128×64  pixels. The frame rate was 500 FPS for subject 1 and 1000 FPS for subjects 2 to 7. The exposure time was 700  μs. We captured 10 videos of the reflected speckle patterns per category using a defocused camera. Each video recording lasted 10 s and started with a 1-s delay after speaker activation to ensure a stable brain cortex reaction.^35^ In this way, we recorded 100,000 frames per category per sample (50,000 for subject 1). The frames were subdivided into chunks (40 frames per chunk, resulting in 2500 chunks per category per subject).

Considering the high sensitivity of our sensor, to ensure that the obtained result was not related to possible head vibration caused by speech-related sound waves, we repeated the measurements after directing the laser to a random area of the participant’s forehead. The data were further used to train and validate the developed DNN model.

### 2.5 Data Processing

#### 2.5.1 Algorithm

The speckle pattern videos, collected from a specific area on the scalp illuminated by a laser beam, contain information about the brain’s response to stimuli based on hemodynamic changes in brain activity. Laser-induced light that penetrates the tissue and is reflected from the skin is affected by brain activity, causing variations in the reflected speckle patterns. For speech comprehension analysis, the laser is directed at the scalp above Wernicke’s area of the brain cortex, which is responsible for speech comprehension. The recorded videos are analyzed using a DNN [Fig. 2(b)].^46^^47^^–^^48^ We frame the task as a binary classification problem with a binary cross-entropy loss, giving probabilities of the input from one of the two classes: clear or incomprehensible language. The input data for the network are a chunk of a speckle pattern video with an arbitrarily selected number of frames per chunk.

We used a ConvLSTM-based architecture with 60,275 trainable parameters. The videos were divided into chunks of 40 frames each. The frame resolution was scaled down to 32×32  pixels, grayscale, to reduce the resulting chunk size in the graphics processing unit (GPU) memory and to save training time. The training was performed on A100 and V100 GPUs with a batch size of 1000 for the two cases. The models were selected based on the accuracy of the validation sets’ performance. Given the balanced data for the two classes, we used accuracy as a metric for model selection. A binary cross-entropy loss was used as a standard for binary classification problems, and the Adam optimizer was chosen because of its adaptable learning rate, which has proven effective in achieving fast convergence. The rectified linear unit (ReLU) activation function was selected for its simplicity and good performance in overcoming the vanishing gradient problem in dense layers. Various regularization techniques, such as batch normalization, dropout, and regularization with the L2 norm, were used.^43^^,^^44^ The collected data were split into training, validation, and test sets.

The first experiment was conducted as follows: 10 videos per person in each category (English/Swedish) were recorded in the morning, and five videos per person in each category (English/Swedish)—in the afternoon for subjects 1 to 7. The training and validation sets consisted of videos from the morning. The test sets comprised the afternoon videos. A total of 80%/20% of the morning videos were divided between the training and validation sets. No video from the morning test was present in the test set. We trained a specific model for each person and evaluated its effectiveness in classifying videos taken from the same person after several hours.

The second experiment was carried out to ensure that the brain cortex response was detected. Similarly, we recorded the speckle patterns from the forehead of one randomly selected subject using the same setup and stimulation. The data were analyzed using the same DNN. The results of the two experiments were compared.

The third experiment tested the ability of the model to generalize to unseen subjects. Although a larger and more representative number of subjects is required for proper generalization to unseen people, we tested the potential for such generalization by creating five different splits (for cross-validation) into training, validation, and test sets. No test set contained data from the training or validation sets of a corresponding split.

#### 2.5.2 Metrics

The experiment was designed to avoid data imbalance between the two tested classes to ensure a reliable accuracy metric. The area under the curve (AUC), F1 score, and Cohen’s kappa coefficient were also used to evaluate the results. The AUC evaluates the performance across all possible classification thresholds rather than at a particular threshold that determines the accuracy.^45^ The receiver operating characteristic (ROC)–AUC curve is often used to determine the threshold balancing between sensitivity and specificity in a way best suited to the problem. Let us define the basic terms TP, TN, FP, and FN and then formulate the metrics of interest, designating the true labels by yi

$$\text{True Positive}\;(\mathrm{TP})=\underset{i}{\sum }(p(xi)==1)\;\;\text{and}\;\;(yi==1),$$ (10)

$$\text{True}\text{ Negative}\;(\text{TN})=\underset{i}{\sum }(p(xi)==0)\;\;\text{and}\;\;(yi==0),$$ (11)

$$\text{False}\text{ Positive}\;(\text{FP})=\underset{i}{\sum }(p(xi)==1)\;\;\text{and}\;\;(yi==0),$$ (12)

$$\text{False}\text{ Negative }(\text{FN})=\underset{i}{\sum }(p(xi)==0)\;\;\text{and}\;\;(yi==1).$$ (13)

The metrics of interest can be calculated as follows:

$$\text{Precision}=\frac{\mathrm{TP}}{\mathrm{TP}+\mathrm{FP}}=\frac{\mathrm{TP}}{\text{Total predicted positive}},$$ (14)

$$\text{Recall}=\frac{\mathrm{TP}}{\mathrm{TP}+\mathrm{FN}}=\frac{\mathrm{TP}}{\text{Total actually positive}},$$ (15)

$$\text{Accuracy}=\frac{\mathrm{TP}+\mathrm{TN}}{\mathrm{TP}+\mathrm{TN}+\mathrm{FP}+\mathrm{FN}}=\frac{\text{correct predictions}}{\text{all predictions}},$$ (16)

$$F1\;\text{score}=\frac{2*\text{Precision}*\text{Recall}}{\text{Precision}+\text{Recall}},$$ (17)

$${\text{Cohen}}^{\prime }s\;\text{kappa for}\;2\;\text{class classification}=\frac{2(\mathrm{TP}*\mathrm{TN}-\mathrm{FN}*\mathrm{FP})}{\left(\mathrm{TP}+\mathrm{FP})*(\mathrm{FP}+\mathrm{TN})+(\mathrm{TP}+\mathrm{FN})*(\mathrm{FN}+\mathrm{TN}\right)}.$$ (18)

Cohen’s kappa (K) metric ranges from −1 to 1. A value of 1 indicates perfect agreement, 0 indicates agreement due to a chance, and −1 indicates complete disagreement. This metric evaluates the level of agreement between predicted and actual classifications and is frequently used to assess the performance and reliability of machine learning classifiers. Cohen’s kappa is typically considered a more robust metric than accuracy, especially for imbalanced datasets or those where it is difficult to assess the expected agreement by chance^45^^,^^49^

$$K=\frac{\text{observed}\_\text{agreement}-\text{probability}\_\text{of}\_\text{agreement}\_\text{by}\_\text{chance}}{\text{maximal}\_\text{possible}\_\text{disagreement}}=\frac{P_{0}-P_{e}}{1-P_{e}}.$$ (19)

By subtracting the probability of agreement by chance (Pe) from the observed agreement (Po) and normalizing by the maximum possible disagreement (1 − Pe), we obtain a measure of agreement that corrects for a chance agreement.

It is essential to emphasize that all experiments were conducted in strict adherence to established guidelines and regulations.

## 3 Experimental Results

### 3.1 Results per Subject

**Fig. 3:**
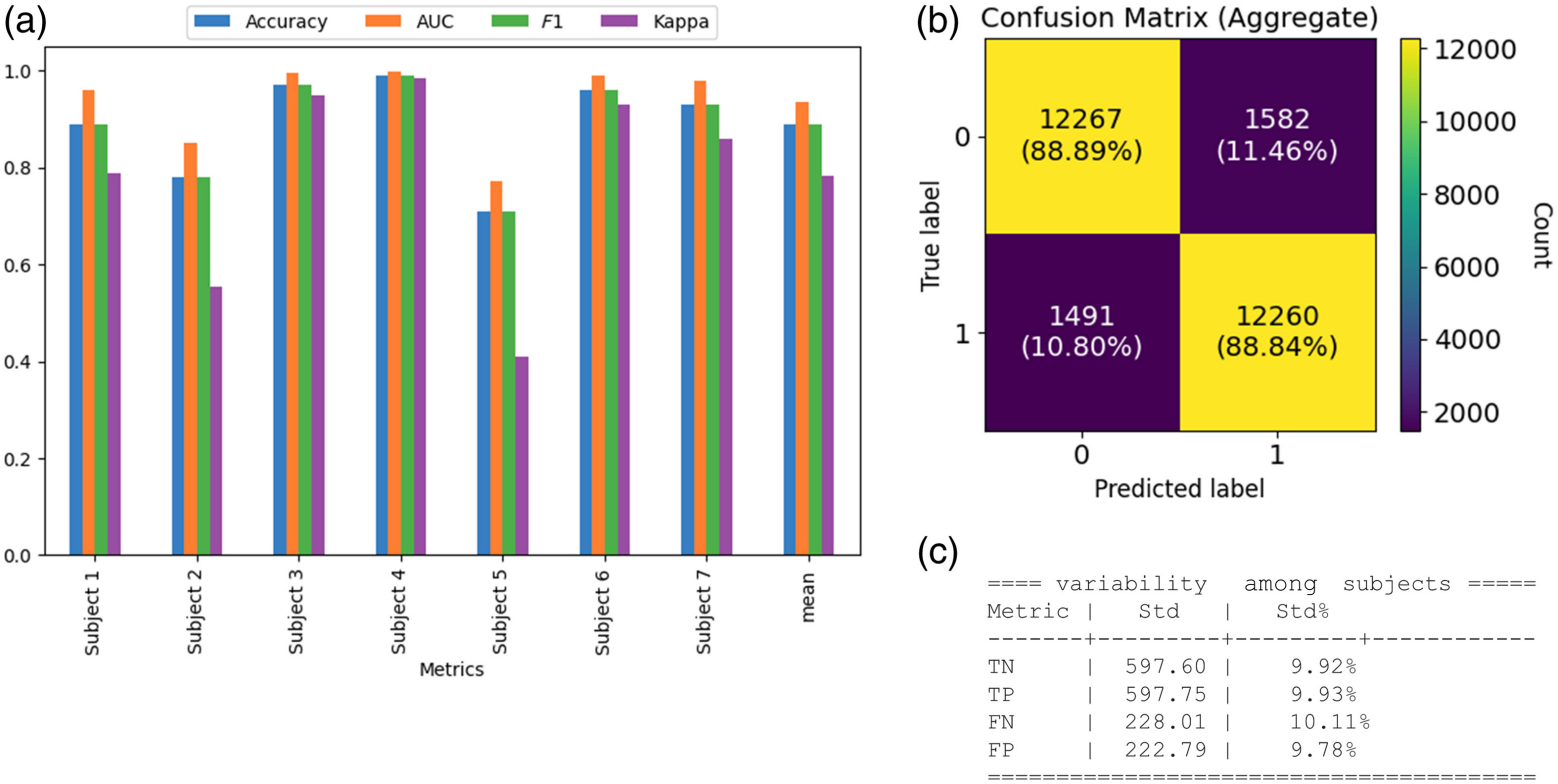
Experiment 1. (a) Metrics for each subject, including their mean. The accuracy mean was 88.89%, the AUC mean was 0.94, the F1 score mean was 0.89, and Cohen’s kappa mean was 0.78. (b) Average confusion matrix (on test) for the first experiment (on 27,600 chunks collected from seven subjects). (c) Variability in TP, TN, FP, and FN results among subjects.

The first experiment aimed to detect and differentiate between feedback from Wernicke’s area of the human brain cortex in response to clear and incomprehensible speech stimuli. The results demonstrated an accuracy of 71% to 99% across all tested subjects, with a mean of 89%. The area under the ROC curve (AUC) ranged from 0.77 to 0.999, with a mean of 0.94, and F1 scores ranged from 0.71 to 0.99. Five models were developed, one per subject, and trained on the morning sample, with the afternoon sample utilized to test the classification quality on the same subject [Table 1, Fig. 3(a)]. The variance across individual models was within 10.11%, as shown in Fig. 3(c). This consistency demonstrates robust performance across participants.

**Table 1:** Results (on test set) of the classifier for the first experiment, including metrics per subject. The batch size was 1000, and the training was performed with an A100 GPU.

Subject #	# epochs	# chunks in the test set (40 frames per chunk)	Cohen Kappa	F1	Accuracy (%)	AUC	Had MRI scan
1	170	2500	0.79	0.89	89	0.96	No
2	240	2500	0.55	0.78	78	0.85	No
3	100	5000	0.95	0.97	97	0.996	No
4	340	2500	0.99	0.99	99	0.999	No
5	100	5000	0.41	0.71	71	0.77	Yes
6	50	5100	0.93	0.96	96	0.99	Yes
7	450	5000	0.86	0.93	93	0.98	No

**Fig. 4:**
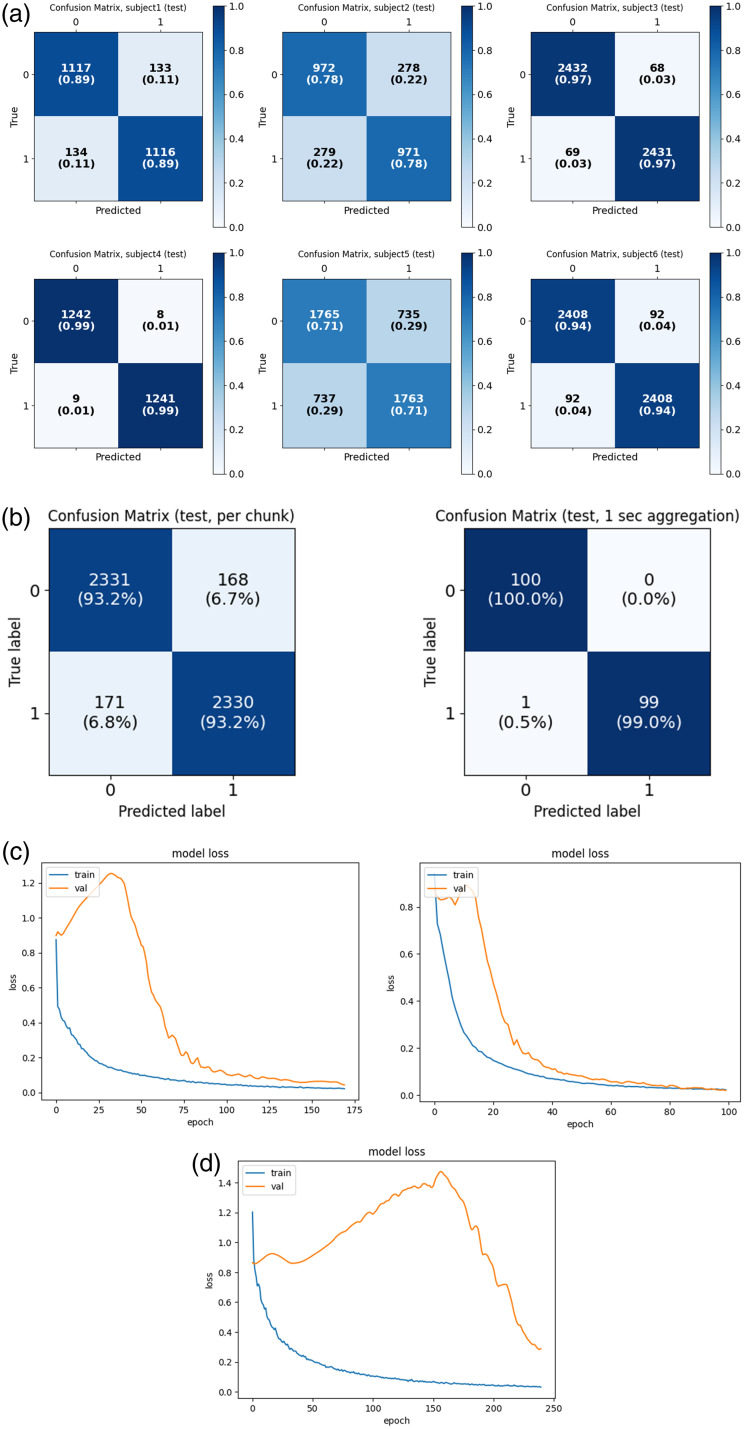
Experiment 1. (a) Confusion matrices for six subjects. (b) A positive effect of aggregation over 1 s. Comparison between nonaggregated (left) and aggregated (right) confusion matrices for subject 7. (c) A typical behavior of the loss function over 464 epochs; validation shown in orange color, while train shown in blue color. The graphs were obtained during 465 the training of perperson models for subjects 1 (left) and 3 (right). (d) For subject 2, training loss curves suggest that 469 additional training epochs could further enhance model performance.

The validation loss [Fig. 4(c), orange line] exhibits a brief initial increase, consistent with early-stage parameter adjustment, followed by a steady decline indicative of stable convergence. Minor oscillations are attributable to regularization methods such as dropout. These dynamics confirm effective model training.

Subject 5, the oldest participant (77 years), exhibited slightly reduced performance, potentially due to age-related factors such as decreased cerebral blood flow and increased difficulty maintaining stillness during recordings—both relevant in a motion-sensitive optical setup. For subject 2, training loss curves suggest that additional training epochs could further enhance model performance [Fig. 4(d)].

**Fig. 5:**
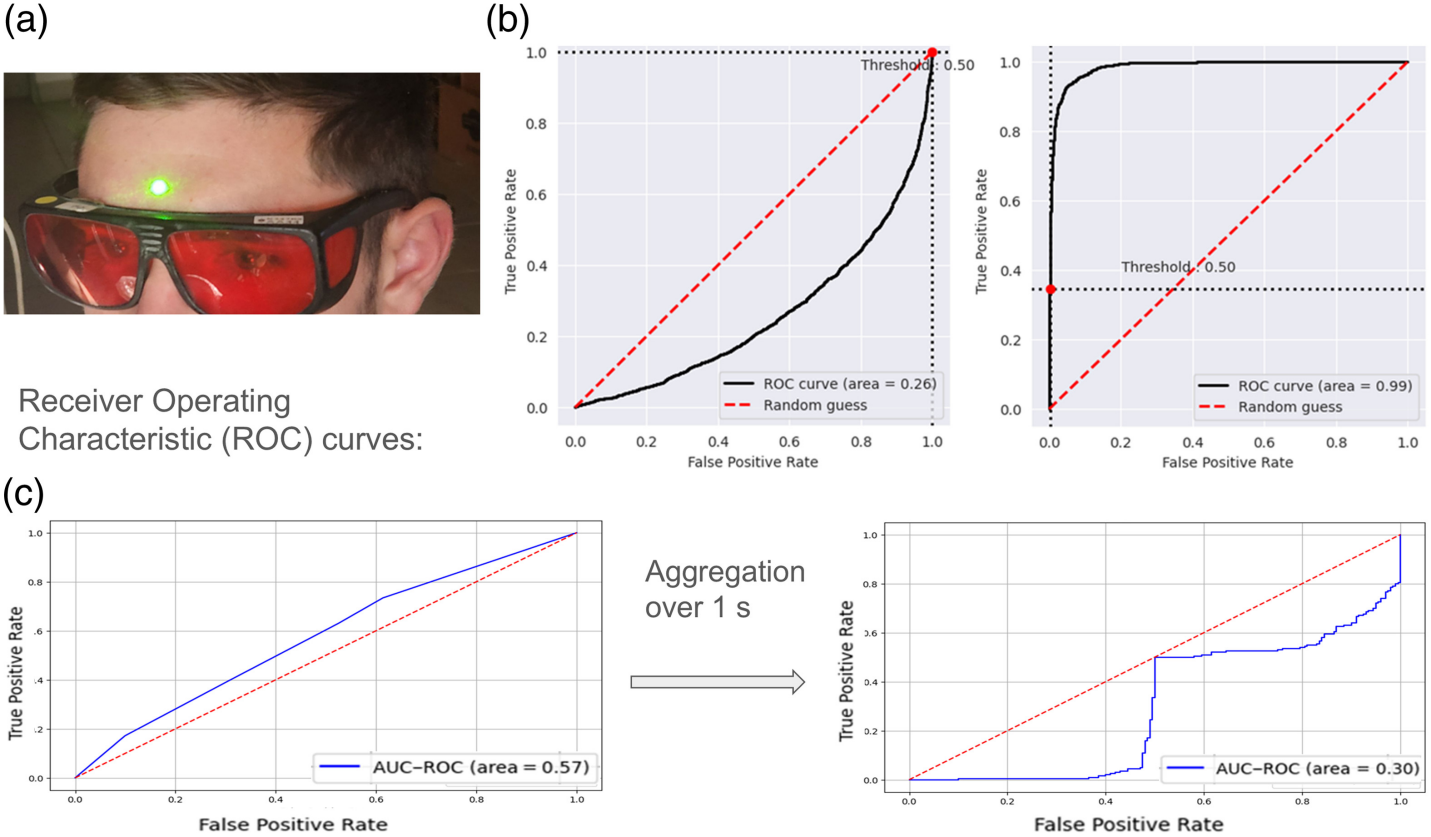
Experiment 2. (a) The green laser spot indicates the forehead location used for the sanity check. (b) AUC–ROC curves for classifying English versus Swedish in subject 5. The left curve (AUC = 0.26) is based on forehead data, whereas the right curve (AUC = 0.99) is based on the data from Wernicke’s area. (c) AUC–ROC curves from an ensemble model (trained on subjects 1 to 6) applied to forehead data from subjects 7 to 8. The left plot shows results without temporal aggregation, and the plot on the right shows results with aggregation over 1 s. Although temporal aggregation significantly improves classification performance for data from Wernicke’s area (see Table 4), it degrades performance on forehead samples of the same subjects.

The second experiment verified the possibility of detecting brain cortex reactions to a clear and incomprehensible speech by capturing speckle patterns from a randomly selected forehead area. Figure 5(a) shows the experimental setup, with a green laser marking the forehead location for data collection. The recordings of speckle patterns, reflected from the forehead of randomly selected subjects under English and Swedish speech stimulation, were analyzed. Samples from the forehead were taken shortly after sampling from the Wernicke area. Such control was implemented to rule out the possibility that the previous classification was based on scalp vibrations related to sound wave pressure. In this case, similar results for both Wernicke’s area and forehead sensing would be obtained. First, we tested the performance of the pretrained model from the first experiment on a sample from the forehead of the same subject (collected on the same day). The classification of the forehead test yielded an AUC of 0.26 [Fig. 5(b), left], an accuracy of 33%, an F1 score of 0.33, and a Cohen’s kappa score of 0.35, indicating that the pretrained DNN that succeeded in classifying the test set from Wernicke’s area (AUC = 0.99, [Fig. 5(b), right]) failed to distinguish between forehead response to English and Swedish. DNN was also trained for the second experiment following a similar protocol: morning forehead samples were used for training and validation, whereas afternoon samples from the same subject’s forehead were used for the test. The classification of the forehead test yielded an AUC of 0.19, an accuracy of 27%, an F1 score of 0.273, and a Cohen’s Kappa score of −0.46, indicating the inability of the DNN to distinguish between forehead response to English and Swedish stimuli. The observed deviation from random chance, particularly for the 40 ms chunks, may be explained by the typical pause length in English and Swedish speech. Finally, an ensemble model trained on data from subjects 1 to 6 was tested on unseen subjects 7 and 8. Although it performed excellently for Wernicke’s area (Table 4), the forehead data showed poor results, with temporal aggregation further degrading performance [Fig. 5(c)]. These findings support the hypothesis that the initial test results obtained from Wernicke’s area reflect the brain cortex response to clear and incomprehensible speech.

### 3.2 Generalization

Despite the limited sample size in the training set, the potential for generalization to unseen subjects (those not included in the model training) was evaluated. Testing the trained model on these unseen subjects demonstrated a generalization capability with an AUC mean of 0.8. (Table 2). Generalization tests were conducted using five distinct splits, each comprising data from two subjects in the training set, two in the validation set, and two in the test set for cross-validation. Data from subjects 1 to 6 was included in the train set and in the test set at least once. Morning and midday samples were analyzed for all subjects. The results were obtained by dividing each test set into 40-frame chunks.

**Table 2:** Generalization of five sets of two subjects in the training, two in the validation, and two in the test sets.

Subjects in split			AUC (test), per chunk	AUC (test), aggregated over 1 s, 25 chunks of 40 frames	Split #
Test	Validation	Train			
3, 6	1, 4	2, 5	0.95	1	1
4,5	2, 3	1, 6	0.67	0.99	2
1, 2	5, 6	3, 4	0.6	0.75	3
5, 6	3, 4	1, 2	0.96	1	4
3, 4	5, 6	1, 2	0.81	0.94	5

Video samples from subjects 2 to 6 were recorded at 1000 fps, whereas the video sample from subject 1 was recorded at 500 fps. The ability of the DNN to generalize across two different frame rates suggests the potential for temporal data augmentation.

**Fig. 6:**
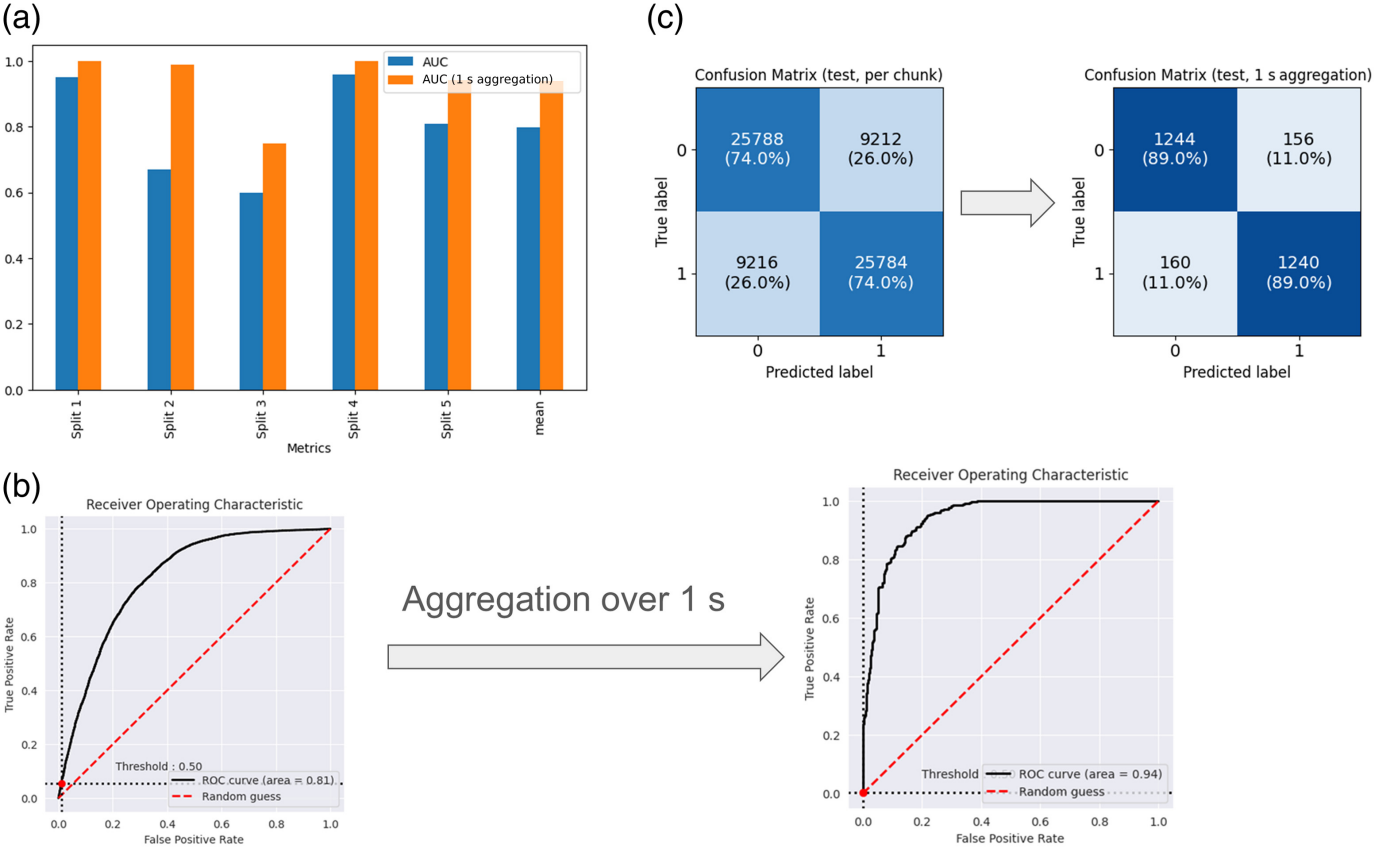
Experiment 3. (a) Comparison of the AUC with and without aggregation for generalization splits 1 to 5. The mean AUC, when calculated per 40-frame chunk, was 0.8. However, when aggregated over 1 s of input, the mean AUC is 0.94. (b) Comparison of AUC—ROC curves of generalized results per chunk (on the left) with AUC of 0.81 versus the 1-s aggregation (of generalized) results with AUC of 0.94 for split 5 (see Table 2). (c) Comparison of confusion matrices of generalized results per chunk (on the left) on 70,000 chunks with 40 frames in each versus the 1-s aggregation (of generalized) results, giving 2800 chunks of 1000 (=40 × 25) frames in each for split 5 (see Table 2).

Given the high recording speed of 1000 fps, a 40-frame chunk represents only a $\frac{1}{25}=0.04$ of a second of the recording for subjects 2 to 6. When predictions were accumulated over 1 s of the recording (summing predictions of 25 consecutive chunks from the same sample and subsequently calculating the AUC), the mean AUC increased to 0.94 (Table 2). Prediction aggregation was performed independently for each input file (10 s per input file) in this test. Figures 6(a) and 6(b) present a comparative analysis of the AUC and Fig. 6(c) compares confusion matrices for one split (split 6), calculated both per chunk and on chunk aggregations.

Figure 6(a) compares the AUCs with and without aggregation across all five splits, demonstrating that aggregation elevates the AUC to 0.94. Additional metrics further corroborate the advantages of aggregation (Table 3). The generalization observed in this limited subject pool suggests that replicating this experiment with a larger, more diverse group will likely enhance production-level generalization.

**Table 3:** Generalization. Comparison of the metrics (on the test set) for one of the splits (split 5, where subjects 1 and 2 were in the train set, subjects 5 and 6 in the validation set, and subjects 3 and 4 in the test set) with and without aggregation. Here, aggregation means accumulating predictions over 1 s of the recording (summing up predictions of 25 consecutive chunks from the same sample and then calculating the metrics).

Aggregated	AUC	Accuracy (%)	F1 score	Cohen Kappa	Split #
No	0.81	74	0.74	0.48	5
Yes	0.94	86	0.86	0.72	

Data collected from subjects 7 and 8 was excluded from the training and validation sets corresponding to generalization splits 1 to 5. These data were subsequently used to evaluate an ensemble model constructed by averaging the outputs of models trained on these splits. The ensemble model achieved an average AUC of 0.93 when the DNN was presented with short data segments (0.25-s speech recordings). Notably, the DNN perfectly distinguished between the two classes when provided with at least 1 s of input (Table 4). However, the optimal thresholds varied across subjects and recording sessions due to differences in lighting conditions and camera gain during video recordings. Though the pretrained models successfully clustered inputs from different classes for unseen subjects, achieving out-of-the-box usability without calibration for individual subjects and recording conditions requires the automatic learning of optimal thresholds. To enable this, a more extensive dataset encompassing diverse conditions and subjects should be collected.

**Table 4:** Generalization. The ensemble model, created by averaging the outputs of five models corresponding to the splits in Table 2, was tested on two new subjects: subjects 7 and 8.

AUC (test) per chunk	AUC (test), aggregated over 1 sec, 25 chunks of 40 frames	Test set
0.881	1	Subject 7, midday test
0.987	1	Subject 8, midday test

## 4 Discussion

The experiments were designed to distinguish responses of the human brain cortex, specifically Wernicke’s area, to comprehensible (English) and incomprehensible (Swedish) speech using AI analysis of the captured speckle pattern videos.

The observed variance within 10.11% across seven individual models (experiment1) demonstrates consistent classification performance, suggesting that the methodology is reliable across diverse subjects. This low variability supports the potential for generalization to broader populations and provides further validation with a larger and more diverse sample pool.

The experiments on eight healthy male subjects of diverse ages (experiment 3) demonstrated initial generalization to previously unseen individuals. We trained models using five data splits from the first 6 participants, with two participants allocated to training, two to validation, and two to testing. The models achieved a mean AUC of 0.94 for classifications based on at least 1 s of input. The ensemble model, which averaged predictions from these five individual models, demonstrated robust performance: when tested on participants 7 and 8, it achieved an AUC of 0.93 for 40-ms (1/25 s) input segments. Notably, aggregating predictions over 1-s intervals improved classification performance to an AUC of 1.0.

Moreover, it was found that processing the speckle patterns captured from different cortex areas, including the forehead (experiment 2), does not allow detection of a difference in the brain response to a clear and incomprehensible speech.

The proposed method for detecting human brain activity presents a cost-efficient alternative to conventional techniques such as fMRI. This approach requires only a laser, a digital camera, and a computer, providing a simplified and accessible solution for brain activity monitoring. The affordability of our method reduces the entry barrier for both research and clinical applications, broadening its potential use.

The simplicity and portability of the method further enhance its utility, allowing its application as a home-care device or integration into medical-grade hospital equipment. The flexibility of video processing—performed on-premises or in the cloud—aligns with the growing demand for remote healthcare solutions, supporting applications in telemedicine and remote neurological assessments. In addition, the portable and contactless nature of the technique makes it particularly suited for monitoring brain cortex function in dynamic events, such as sports and fitness activities, further extending its range of applications. However, the method requires extending the tests by involving a representative group of participants for future experiments.

Future research directions may include the following:

1. Dataset expansion and diversity: Although the results demonstrate potential generalization to unseen individuals, a more extensive and diverse dataset is essential to train a DNN capable of robust generalization across varying populations.
2. Though the pretrained models successfully clustered inputs from different classes for unseen subjects, achieving out-of-the-box usability without calibration for individual subjects and recording conditions requires automatic learning of the optimal thresholds. To enable this, the collection of a larger dataset encompassing diverse conditions and subjects is required.
3. Inclusive participant sampling: This study’s participants included only male subjects, which may limit the generalizability of the findings. Future work will aim to include a more diverse cohort to validate the approach across genders. Although the current experiment included participants of varying ages, further studies should investigate the influence of demographic and age-related factors on brain activity detection.
4. Enhanced hardware setup: Developing a head-mounted device incorporating a laser and camera could minimize the impact of head movements, strengthening some of the method’s practical use cases.
5. DNN performance optimization: Exploring and comparing alternative DNN architectures’ pretrained foundation models with the ConvLSTM-based approach used in this study to further improve the performance and robustness of the system.
6. Mitigating distribution shifts: Addressing robustness to distribution shifts among samples will be critical to achieving consistent performance across diverse conditions.
7. Exploring the use of lasers with different wavelengths, such as near-infrared (NIR) light with higher tissue penetration, presents the potential to monitor brain regions within the cortex’s sulci or beyond the cortical surface. However, this approach introduces challenges, including increased light scattering, which may impact signal quality. Addressing these challenges could significantly expand the scope of this research.

This study represents an important step toward establishing speckle pattern analysis as an affordable and noninvasive technique for detecting human brain cortex response. By combining AI-driven analysis with cost-effective hardware, this method offers a promising alternative to traditional brain monitoring systems. Ongoing research efforts will focus on refining the technique, improving generalization, and expanding its applicability across broader scientific and clinical contexts.

## 5 Conclusion

The study introduces a noninvasive approach for remotely detecting human brain activity in Wernicke’s area using speckle pattern analysis enhanced by deep learning. This method employs a laser beam to illuminate a human scalp. It captures the secondary speckle patterns with a defocused camera, demonstrating the feasibility of classifying brain cortex (Wernicke’s area) responses to clear versus incomprehensible speech stimuli. A ConvLSTM-based neural network was utilized to analyze the captured speckle patterns, achieving an average classification accuracy of 89% and a mean AUC of 0.94 across all subjects in per-subject models. The proposed method exhibited robust performance, with individual model variance remaining within 10.11% across subjects. Experimental results of experiments conducted on eight participants confirm the system’s capability to differentiate between the brain’s response to clear and incomprehensible speech. The tests prove that the detected signals originated from brain activity rather than external vibrations or scalp-related artifacts.

Moreover, the method demonstrated notable generalization potential, achieving a mean AUC of 0.80 for unseen subjects when DNN input contained short data chunks (corresponding to 1/25 s of speech recording), which increased to 0.94 with aggregation over 1-s recording intervals. The classifier performance further improved when the ensemble model was used. This highlights the system’s potential for real-world applications after training on a more extensive and diverse dataset.

The proposed method offers significant advantages compared with existing brain activity monitoring technologies. It is affordable, portable, and contactless, requiring only a laser, camera, and computer. These features make it a practical and cost-effective solution for dynamic environments, including sports and real-life activities. The system’s contactless nature also makes it well-suited for scenarios where physical scalp contact is undesirable or impractical, such as for individuals with sensory sensitivities.

Finally, this research highlights the potential of the photonic speckle pattern analysis as a transformative tool for remote brain activity monitoring. By addressing key limitations of the current technologies, this approach paves the way for noninvasive, accessible, and versatile solutions for brain research and diagnostics. Future work should aim to expand the subject pool and explore additional use cases to enhance the system’s generalization capabilities and deployment readiness. Reducing the need for per-patient calibration will further optimize the system’s applicability and reduce operational time. The work continuation could also include distinguishing between the brain reaction to music, noise, speech, and other brain stimuli as well as distinguishing between the brain reaction to speech in an incomprehensible and native language when native languages differ among participants.

## Data Availability

The data generated to support the findings of this study are available from the corresponding author upon reasonable request. The code is available at https://github.com/natalyasegal/SpecklesAI.
